# Insights Into the Evolution of *Staphylococcus aureus* Daptomycin Resistance From an *in vitro* Bioreactor Model

**DOI:** 10.3389/fmicb.2019.00345

**Published:** 2019-02-28

**Authors:** Erica Lasek-Nesselquist, Jackson Lu, Ryan Schneider, Zhuo Ma, Vincenzo Russo, Smruti Mishra, Manjunath P. Pai, Janice D. Pata, Kathleen A. McDonough, Meenakshi Malik

**Affiliations:** ^1^Wadsworth Center, New York State Department of Health, Albany, NY, United States; ^2^Albany College of Pharmacy and Health Sciences, Albany, NY, United States; ^3^Department of Biomedical Sciences, University at Albany, School of Public Health, Albany, NY, United States; ^4^Department of Clinical Pharmacy, University of Michigan, Ann Arbor, MI, United States

**Keywords:** *Staphylococcus aureus*, bioreactor culture, daptomycin, whole-genome sequencing analysis, evolution of resistance

## Abstract

The extensive use of daptomycin for treating complex methicillin-resistant *Staphylococcus aureus* infections has led to the emergence of daptomycin-resistant strains. Although genomic studies have identified mutations associated with daptomycin resistance, they have not necessarily provided insight into the evolution and hierarchy of genetic changes that confer resistance, particularly as antibiotic concentrations are increased. Additionally, plate-dependent *in vitro* analyses that passage bacteria in the presence of antibiotics can induce selective pressures unrelated to antibiotic exposure. We established a continuous culture bioreactor model that exposes *S. aureus* strain N315 to increasing concentrations of daptomycin without the confounding effects of nutritional depletion to further understand the evolution of drug resistance and validate the bioreactor as a method that produces clinically relevant results. Samples were collected every 24 h for a period of 14 days and minimum inhibitory concentrations were determined to monitor the acquisition of daptomycin resistance. The collected samples were then subjected to whole genome sequencing. The development of daptomycin resistance in N315 was associated with previously identified mutations in genes coding for proteins that alter cell membrane charge and composition. Although genes involved in metabolic functions were also targets of mutation, the common route to resistance relied on a combination of mutations at a few key loci. Tracking the frequency of each mutation throughout the experiment revealed that mutations need not arise progressively in response to increasing antibiotic concentrations and that most mutations were present at low levels within populations earlier than would be recorded based on single-nucleotide polymorphism (SNP) filtering criteria. In contrast, a serial-passaged population showed only one mutation in a gene associated with resistance and provided limited detail on the changes that occur upon exposure to higher drug dosages. To conclude, this study demonstrates the successful *in vitro* modeling of antibiotic resistance in a bioreactor and highlights the evolutionary paths associated with the acquisition of daptomycin non-susceptibility.

## Introduction

*Staphylococcus aureus* is one of the pathogens most commonly associated with both community and hospital acquired infections (Chambers and DeLeo, 2009; Leclercq, 2009; Patel, 2009). Antibiotic management of infections caused by *S. aureus* is complicated by the emergence of methicillin resistant *S. aureus* (MRSA), vancomycin intermediate *S. aureus* (VISA), and vancomycin resistant *S. aureus* (VRSA) (Chambers and DeLeo, 2009; Howden et al., 2010; Bayer et al., 2013). More recently, daptomycin nonsusceptible (according to Clinical Laboratory Standards Institute 2016 guidelines) strains of *S. aureus* (herein referred to as DAP resistant or DAP-R) have emerged as daptomycin increasingly becomes the drug of choice for infections caused by MRSA and VISA strains (Marty et al., 2006; Skiest, 2006; Fowler et al., 2007; Jones et al., 2008; Stryjewski and Corey, 2014).

Previous studies have described the acquisition of DAP-R by *S. aureus* as a complex multifactorial process (Chen et al., 2015; Tran et al., 2015; Kang et al., 2017), with contributions from expression changes and mutations in genes involved in cell wall and cell membrane homeostasis (Friedman et al., 2006; Camargo et al., 2008; Yang et al., 2009; Fischer et al., 2011; Patel et al., 2011; Bayer et al., 2014, 2015; Capone et al., 2016; Kang et al., 2017; Ma et al., 2018; Sabat et al., 2018). The majority of these studies characterized transcriptional changes and/or genetic mutations in clinical DAP-R isolates of *S. aureus* that emerged as a result of prolonged treatment with (1) daptomycin alone, (2) other antibiotics (typically vancomycin) followed by the administration of daptomycin, or (3) other antibiotics with no daptomycin treatment (Friedman et al., 2006; Murthy et al., 2008; Mishra et al., 2009, 2011, 2014; Yang et al., 2009; Howden et al., 2010; Boyle-Vavra et al., 2011; Fischer et al., 2011; Cameron et al., 2012; Hafer et al., 2012; Peleg et al., 2012; Bayer et al., 2014, 2015; Cafiso et al., 2014; Berti et al., 2015; Gaupp et al., 2015; Capone et al., 2016; Kang et al., 2017; Sabat et al., 2018). Serial passaging of *S. aureus* on plates or liquid culture by exposing the bacteria to fixed concentrations of antibiotic and identifying the associated molecular and/ or phenotypic changes represents another strategy employed to elucidate the mechanisms of daptomycin resistance (Silverman et al., 2001; Friedman et al., 2006; Mishra et al., 2009, 2012; Patel et al., 2011; Peleg et al., 2012; Berti et al., 2015; Müller et al., 2018). While these studies identify targets of mutation associated with killing by antibiotics, they only provide a snapshot of the evolutionary process after all mutations have been acquired. In the case of clinical isolates there is no knowledge of which mutations accumulate in response to specific levels of treatment while *in vitro* studies involving serial passage often characterize the response to only a single dose of antibiotic or describe the final DAP-R strain. Similarly, mutations selected in response to vancomycin treatment or other antibiotics do not necessarily represent predominant mutations that would emerge after daptomycin treatment alone. It has been reported that *S. aureus* strains can develop vancomycin heteroresistance which makes them DAP-R despite never having been exposed to daptomycin (Pillai et al., 2007). Therefore, isolates of *S. aureus* that were resistant to other antibiotics prior to acquisition of DAP-R do not provide the details of DAP-R evolution in a DAP-S population. Better understanding of the evolution of DAP-R in *S. aureus* would derive from a system, such as a bioreactor, where a DAP-S bacterial population grown at a steady state is exposed to increasing concentrations of daptomycin, with mutations responsible for daptomycin resistance identified at each step (Toprak et al., 2011, 2013).

The goals of our study were to establish the bioreactor as a tool for realistically modeling the emergence of drug resistance and to use the bioreactor to further elucidate the evolution of daptomycin resistance in susceptible *S. aureus* populations by overcoming some of the limitations of alternative methods. Our results demonstrate that a continuous culture bioreactor maintains bacterial growth at a steady state while also maintaining the concentration of daptomycin at desired levels. We recovered single nucleotide polymorphisms (SNPs) in genes previously implicated in DAP-R as well as mutations in possible new targets of resistance. In comparison, the serial passaged culture showed only one mutation potentially associated with daptomycin resistance and did not offer the level of insight into mutation frequency fluctuations that the bioreactor populations provided. Our bioreactor results support the hypothesis that resistance at higher daptomycin concentrations requires only a few key changes and that these mutations often emerge much earlier in the history of the population, underscoring the need to examine the path to high minimum inhibitory concentrations (MICs), not just the endpoint.

## Materials and Methods

### Bacteria Parental Strain

*Staphylococcus aureus* strain N315 (ATCC 29213) obtained from BEI Resources (Catalog No. NR-45898), Manassas, VA, United States (Biodefense and Emerging Infections Research Resources)^1^ served as the parental DAP-S strain and was propagated in Mueller Hinton Broth (MHB) (BD BBL^TM^) and frozen in 15% glycerol in 1 ml aliquots for further use. Daptomycin resistant isolates were derived from the parent N315 strain which was consistently exposed to increasing doses of daptomycin in MHB in a bioreactor or in serial passaged flask culture. For every experiment, blood agar plates (Trypticase Soy Agar II with 5% sheep blood, BD Biosciences) were streaked with bacteria from frozen glycerol stocks. After overnight incubation at 37°C with 5% CO_2_, a single colony was used to seed the bioreactor or flask culture populations. Three bioreactor replicates (populations A–C) and one serial passaged flask culture experiment (population P) were performed with populations B and C being exposed to 0, 6, 10, and 14 μg/ml daptomycin and populations A and P being exposed to 6 and 10 μg/ml of daptomycin.

### Daptomycin

Clinical grade Cubicin^®^ (injectable daptomycin) from a single lot was purchased from Albany Medical Center Outpatient Pharmacy, Albany NY, United States. The daptomycin was supplied as 500 mg vials and was reconstituted in sterile water to achieve desired concentrations to be used in various experiments.

### Serial Passage Flask Culture

Bacterial flask cultures were grown overnight in MHB supplemented with 50 mg/L calcium and 12.5 mg/L magnesium and adjusted to 10^7^ CFU/ml concentration. This served as the 0-h time point. After two hours, the concentration of bacteria was roughly 10^8^ CFU/ml at which time 6 μg/ml daptomycin was added. Both treated and untreated flasks were grown overnight and subsequently plated on 6 μg/ml daptomycin drug plates. Colonies were isolated, grown overnight, and frozen for further analysis. This process was repeated at 10 μg/ml daptomycin with the colonies from 6 μg/ml drug exposure serving as the seed populations.

### Bioreactor Culture and Adaptation Conditions

The bioreactor system is a continuous culture system in which the rate of inflow (fresh medium) and outflow (spent medium containing culture) is equal, resulting in a constant volume of 400 ml culture in the vessel. The system maintains the bacterial population in exponential growth at a constant density (except after initial additions of antibiotic), as confirmed by CFU counts and optical densities (ODs) of cultures sampled at different time points. MHB was inoculated with a single colony of *S. aureus* N315 strain and maintained for a period of 14 days at a constant OD_600_ of 0.045 (equivalent to 1 × 10^7^ CFU/ml) in a 400 ml volume by automated dilution of the culture from feed reservoirs in a Sartorius Stedim Biostat A plus bioreactor. Briefly, thawed glycerol stock of N315 was streaked out on blood agar plates. A single colony was picked to inoculate 20 ml of MHB, which was incubated overnight in an orbital shaker at 37°C. The next day, the inoculum was diluted 1:1 to make a 40 ml starting culture of 0.4 OD_600_. The starting culture was added to 360 ml in the bioreactor and then maintained at an OD_600_ of 0.45. Daptomycin was added in step-wise increments at a concentration of 6 μg/ml after 24 h, 10 μg/ml after 120 h, and 14 μg/ml after 220/320 h of growth.

### Isolation of DAP-S and DAP-R Isolates

Samples were collected from the bioreactor at 24, 120, and 220/320 h following exposure to 0, 6, 10, and 14 μg/ml of daptomycin. Samples from population P were collected after exposure to 0, 6, and 10 μg/ml of daptomycin after 24 h of growth. Stocks of both population-based and single colony-based resistant isolates were made in 15% glycerol and stored at -80°C for further analysis. Colonies were isolated by streaking Mueller-Hinton agar plates supplemented with 50 mg/ml calcium and 6 μg/ml daptomycin.

### Measurement of Bacterial Growth Rate

Isolates were grown in a shaking incubator at 37°C. Aliquots were collected at 2 h intervals for a period of 6–8 h for populations A–C. Samples from population P were collected at 0, 2, 4, 6, and 24 h starting from the 0-h time point. The aliquots were serially diluted and plated on sheep blood agar plates to quantify bacterial numbers. The plates were incubated at 37°C overnight. The colonies were counted and the results were expressed as log_10_ CFU/ml.

### Antimicrobial Susceptibility Studies

The MIC values were determined for isolates using either Epsilometer test (E test) or broth microdilution method. The MIC was considered to be the lowest concentration of daptomycin required to inhibit the growth of *S. aureus* N315. All susceptibility testing was performed using samples in triplicate and each experiment was repeated at least twice. For E tests, bacterial cultures of isolates were grown to OD_600_ of 0.08–0.09 in 5 ml cation-adjusted MHB (CAMHB) containing 25 mg/ml calcium and 12.5 mg/ml magnesium. Sterile cotton swabs were used to uniformly streak a lawn of each sample on trypticase soy agar plates containing 5% sheep blood. The cultures were allowed to be completely adsorbed. Using sterile tweezers, the E test strips containing varying concentrations of daptomycin (BioMérieux Inc.) were placed on the streaked agar plates. The plates were incubated at 37°C for 18–20 h. The elliptical zones of inhibitions were read to determine the MIC values.

The broth dilution method was performed according to the Clinical Laboratory Standards Institute (CLSI) guidelines. Blood agar plates streaked with bioreactor-derived isolates and a control N315 strain were grown overnight. Single colonies were picked, inoculated in 5 ml CAMHB and grown to achieve an OD_600_ of 0.08–0.09, which corresponds to 1 × 10^8^ CFU/ml. Daptomycin with a starting concentration of 32 μg/ml was serially diluted two-fold in a sterile 96-well plate to achieve a final concentration of 16, 8, 4, 2, 1, 0.5, 0.25, and 0 μg/ml. In accordance with the CLSI standards, bacterial suspension diluted to a final concentration of 5.5 × 10^5^ CFU/ml was added to each well of the plate containing varying concentrations of daptomycin. The 96-well plate was incubated for 24 h after which the results were recorded.

### DNA Extraction and Whole Genome Sequencing

Bacterial pellets from overnight cultures of the reference *S. aureus* N315 strain, bioreactor-derived isolates and colonies, and serial passaged isolates and colonies were treated with lysostaphin and lysozyme to disrupt the peptidoglycan layer of the cell wall. Genomic DNA was purified using PureLink Genomic DNA Mini Kit (Invitrogen) and samples were sent for whole genome DNA sequencing at Wadsworth Center, New York State Department of Health, Albany, NY, United States. Whole genome sequencing libraries were prepared with the Nextera DNA library preparation kit (Illumina) and sequenced using the standard 500 cycle V2 protocol on an Illumina MiSeq. Whole genome sequences were required to have an average depth of coverage of at least 60 × before analysis.

### Identification of Mutations Associated With Daptomycin Resistance

The subroutine BBDuk from BBtools v36.38^2^ quality trimmed raw reads and removed any remaining adaptors with the following parameters: qin = 33, ktrim = r, mink = 11, trimq = 20, minlength = 100, tbp = t, and tpe = t. Sequence libraries for our parent N315 genome and all subsequent populations and clones were reference aligned to the N315 genome from NCBI (Refseq accession number NC_002745.2) with BWA mem v.0.7.5 (Li and Durbin, 2009). SAMtools and BCFtools 0.1.19 (Li and Durbin, 2009; Li, 2011) detected single nucleotide polymorphisms (SNPs) from each reference alignment – considering only base positions with a Phred score > = 20 and reads with a minimum mapping score of 20. We further filtered the list of SNPs for each population by only examining mutations at positions covered by a depth of 20 or more reads and where 5% or more of the reads supported the alternative nucleotide. We considered a SNP to be present in colonies if positions were covered by 20 or more reads and 95% or more of the reads supported the alternative nucleotide. The presence of each potential SNP and its quality were confirmed in IGV v.2.3.78 (Robinson et al., 2011; Thorvaldsdóttir et al., 2013) and mutations that occurred in highly variable regions (such as phage insertions) were discarded. Custom designed Python scripts associated mutations with coding or non-coding regions to identify changes potentially involved in daptomycin resistance and to translate codons into amino acids.

Accession numbers for sequence data generated from this project have been deposited in GenBank under the BioProject PRJNA446060 http://www.ncbi.nlm.nih.gov/bioproject/446060 and SRA accession SRP136646.

## Results

### *Staphylococcus aureus* N315 Acquires Resistance to Daptomycin in a Bioreactor

**FIGURE 1:**
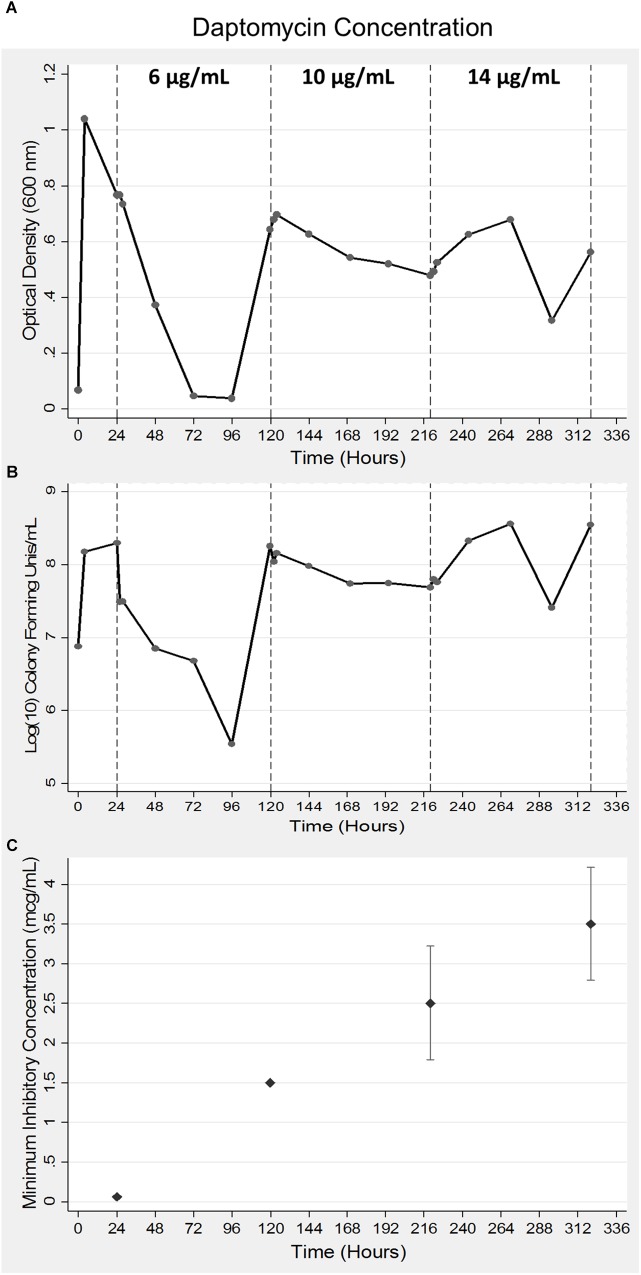
Acquisition of daptomycin resistance in *S. aureus* using a bioreactor model. Bacteria were grown for a period of 24 h after which daptomycin was added at indicated concentrations at 24, 120, and 220 h of growth in a bioreactor. Samples were collected at 24 h intervals to measure optical density at 600 nm **(A)** and bacterial viability was determined by counting CFUs **(B)**. The data shown are representative of three independent experiments conducted **(A–C)**. The MICs of the bacterial aliquots collected at the indicated times were determined as described in the Methods section **(C)**. The data are presented as mean ± SD and are representative of three independent experiments conducted with two replicates each.

A 35-fold reduction in bacterial viability was observed following the addition of 6 μg/ml concentration of daptomycin that was corroborated by a sudden drop in the OD_600_ values (Figure 1A,B). Bacterial numbers returned to the starting concentration of 1 × 10^8^ CFUs within the next 24 h of growth (Figure 1B) at which time the second bolus of daptomycin at 10 μg/ml was added. The second treatment resulted in a moderate 10-fold drop in bacterial viability, which remained at the same level for next 96–120 h of growth (Figure 1B). An identical pattern for OD_600_ values was also observed (Figure 1A). The addition of the third bolus of 14 μg/ml of daptomycin after 220 h of growth did not reduce bacterial viability. On the contrary, a 5–10-fold increase in bacterial numbers was observed (Figure 1B). Corresponding increases in the MIC values were observed in association with enhanced bacterial viability in response to increasing doses of daptomycin (Figure 1C). In summary, the parent *S. aureus* was initially sensitive to the smallest dose (6 μg/ml) of daptomycin but DAP resistant *S. aureus* emerged, which progressively exhibited an enhanced resistance to increasing exposures of daptomycin.

### The Bioreactor Recovers Clinically Relevant Mutations Associated With Daptomycin Resistance

Our parental N315 genome (sequenced after acquisition from BEI but before introduction to the bioreactor or serial passage) deviated from the NCBI N315 strain (NC_002745.2) by eight SNPs, which were present in all bioreactor passaged populations (A–C) and clonal lines (Supplementary Table S1) and the serial passaged population P and its clonal lines (with the exception of the absence of the substitution at position 939485 in population C; Supplementary Table S1).

All three bioreactor populations (A–C) evolved mutations at the phosphatidylglycerol lysyl-transferase *mprF* gene (SA1193) after exposure to daptomycin. Populations B and C also exhibited mutations at the cardiolipin synthase *cls2* locus (SA1891; Table 1). These mutations, as well as the SNPs retrieved from the CDP-diacyclycerol-glycerol-3-phosphate 3-phosphatidyltransferase *pgsA* gene (SA0828) and the PAS sensor histidine kinase *walK* (previously *yycG*) gene (SA0018), have been recorded in clinical or laboratory DAP-R strains (Friedman et al., 2006; Howden et al., 2011; Peleg et al., 2012; Bayer et al., 2015; Chen et al., 2015; Kang et al., 2017). We also detected mutations in the *clpX* gene (SA1498) and the ABC transporter permease gene *vraG* gene (SA0617), which have been shown to vary in response to daptomycin exposure (Cameron et al., 2012; Peleg et al., 2012; Song et al., 2013).

**Table 1:** SNP acquisition in bioreactor and serial passaged *Staphylococcus aureus* populations exposed to daptomycin.

Pop^a^	DAP exposure^c^	Genome position	Sequence type	Amino acid change	Gene name^e^	Protein product^f^
A	6, 10	1278854	Coding	K135E	SA1126/pgsA	CDP-diacylglycerol–glycerol-3-phosphate 3-phosphatidyltransferase
A	6, 10	1364495	Coding	S295L	SA1193/mprF	phosphatidylglycerol lysyltransferase
A^b^	6	2288162	Noncoding	NA	SA_RS11590	hypothetical protein
B	10	26957	Coding	S437F	SA0018/walK	PAS domain-containing sensor histidine kinase
B	10	261590	Coding	N151N	SA0219	pyruvate formate-lyase-activating enzyme
B	6, 10, 14	863920	Coding	Q221^∗d^	SA0756	3-dehydroquinase
B	6, 10, 14	1364495	Coding	S295L	SA1193/mprF	phosphatidylglycerol lysyltransferase
B	6, 10	1364634	Coding	L341F	SA1193/mprF	phosphatidylglycerol lysyltransferase
B	10	1706314	Coding	R364C	SA1498	ATP-dependent Clp protease ATP-binding subunit ClpX
B^b^	10, 14	2143178	Coding	A23V	SA1891/cls2	cardiolipin synthase
B^b^	10	2143266	Coding	L52F	SA1891/cls2	cardiolipin synthase
B	10	2399255	Noncoding	NA	SA2134	DNA-3-methyladenine glycosylase
C^b^	14	382426	Noncoding	NA	SA0325	glycerol-3-phosphate transporter
C	14	525203	Coding	F33V	SA0454	pur operon repressor
C^b^	14	705637	Coding	N320N	SA0610	lipase LipA
C	14	708186	Coding	Q294R	SA0613	3-beta hydroxysteroid dehydrogenase
C^b^	0	710848	Coding	M1R	SA0617/vraG	bacitracin ABC transporter permease
C^b^	14	730798	Noncoding	NA	SA0638	undecaprenyl-diphosphatase
C	6, 10, 14	906481	Coding	G170D	SA0802	NADH dehydrogenase
C	14	1018829	Coding	A223G	SA0897	2-succinyl-6-hydroxy-2,4-cyclohexadiene-1-carboxylate synthase
C	6, 10, 14	1364621	Coding	S337L	SA1193/mprF	phosphatidylglycerol lysyltransferase
C^b^	14	1493152	Coding	D857G	SA1288	ATP-dependent helicase DinG
C^b^	14	1510280	Coding	M25I	SA_RS07380	hypothetical protein
C	6, 10, 14	2143266	Coding	L52F	SA1891/cls2	cardiolipin synthase
C^b^	14	2295261	Coding	C265W	SA2023	DNA-directed RNA polymerase subunit alpha
C^b^	14	2325759	Coding	T106I	SA2062	transcriptional regulator
C^b^	0	2550881	Coding	T142S	SA2275	hypothetical protein
P	10	1278981	Coding	S177F	SA1126/pgsA	CDP-diacylglycerol–glycerol-3-phosphate 3-phosphatidyltransferase
P	10	1295864	Coding	S64I	SA1139	glycerol-3-phosphate-responsive antiterminator
P^b^	10	1300305	Coding	W338^∗d^	SA1142	glycerol-3-phosphate dehydrogenase


^a^Pop, Bioreactor and serial passaged populations; ^*b*^mutations detected in > 5% but < 20% of population as assessed by read depth; ^*c*^Dap exposure, the concentration of daptomycin at which the mutation was recorded to have occurred in the population – 0, 0 μg/ml daptomycin; 6, 6 μg/ml daptomycin; 10, 10 μg/ml daptomycin; 14, 14 μg/ml daptomycin; ^*d^change to a stop codon; ^*e*^Gene abbreviations included with the gene names highlight genes with previously determined associations to DAP resistance in clinical and/or laboratory strains of *S. aureus*; ^*f*^Protein product descriptions for intergenic mutations represent the closest gene to the intergenic SNP.

The bioreactor also recovered new mutations that might be candidates for contributing to daptomycin resistance but require further exploration, including the nonsynonymous substitutions in the genes coding for an ATP-dependent helicase and the *pur* operon repressor and an intergenic mutation near the DNA-3-methladenine glycosylase locus (Table 1). Serial passaged population P showed only three mutations after exposure to 10 μg/ml daptomycin and only the SNP in the *pgsA* gene occurred at a locus previously associated with daptomycin resistance in laboratory strains (Table 1; Peleg et al., 2012).

### Frequency Profiles Reveal Early Appearance of Mutations in Bioreactor Experiments

**FIGURE 2:**
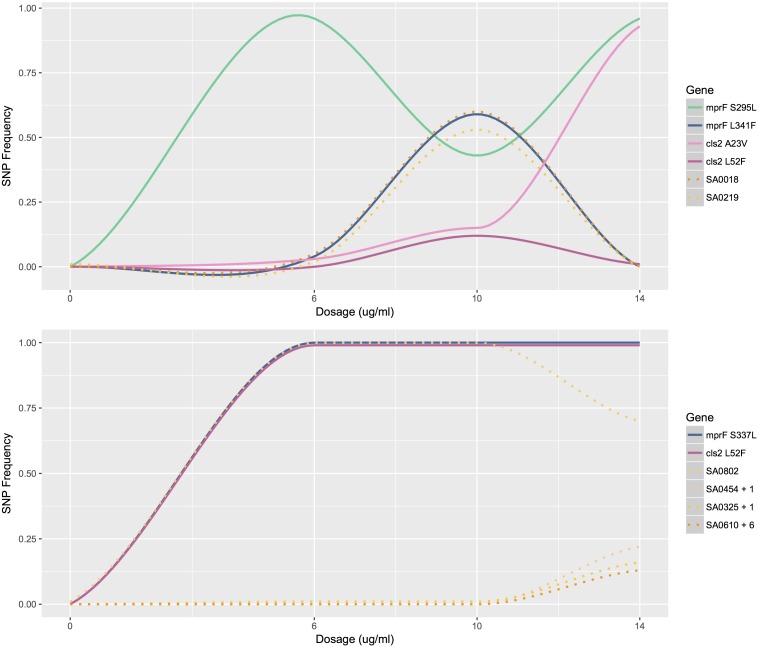
Mutation profiles of populations B and C. The frequency of mutations as a function of antibiotic concentration for population B (top) and C (bottom). Mutations profiled are SNPs that lead to amino acid changes in MprF and Cls2. Other mutations profiled are listed by gene name. SA0454 + 1 represents the similar mutation profiles of SA0454 and SA0613; SA0325 + 1 represents the similar mutation profiles of SA0325 and SA0897; SA0610 + 6 represents the similar mutation profiles of SA0610, SA2134, SA1228, SA_RS07380, SA2023, and SA2062.

Tracking the frequency of mutations throughout the course of the bioreactor experiments revealed that many SNPs appeared earlier than were identified by our filtering criteria (See example: Figure 2). Mutations present at very low frequencies at no or low daptomycin concentrations were often observed reaching high frequencies at 10 or 14 μg/mL daptomycin in population B. This suggests that variants observed in less than 5% of the population, which did not pass our reporting threshold, actually represent real mutants present in earlier populations (Supplementary Table S2. For example, the SNP at position 2143178 (in *cls2*) in population B was present in 3% of the reads at 6 μg/ml daptomycin (Figure 2), but was not recorded at this time point because it did not pass our 5% reporting threshold. However, this SNP increased to 15% at 10 μg/ml and almost reached fixation at 14 μg/ml (represented in 93% of the population; Supplementary Table S2 and Figure 2). Similarly, the SNPs at positions 26957, 261590, 1364495, and 2399255 in population B (in genes *walK*, SA0219, *mprF*, and the noncoding region near SA2134, respectively) reached intermediate frequencies at 10 μg/ml daptomycin but were actually present earlier in < = 5% of population (Supplementary Table S2). Although population C appeared relatively stable until 14 μg/ml, some SNPs were already present at low frequencies at 0, 6, and 10 μg/ml daptomycin concentrations (Supplementary Table S2 and Figure 2). In contrast, serial passaged population P showed no indication of mutations until after the highest drug exposure of 10 μg/ml (Supplementary Table S2. Thus, sampling throughout drug exposures in the bioreactor revealed that mutations relevant to resistance at high antibiotic concentrations often appeared much earlier in the history of the population.

### Colonies Confirm Presence of Low Frequency Variants

The three colonies sequenced from population A at 6 μg/ml and populations B and P 10 μg/ml daptomycin exposure helped to confirm the presence of several SNPs in the populations from which they derived (Supplementary Table S3). Mutations present in the two populations but absent in their colonies most likely reflect the heterogeneous composition of the populations, but could also indicate poor recovery of mutant cells on plates or represent false-positive SNPs. For example, the nonsynonymous mutation in the *pgsA* gene was detected in 68% of the reads in population A at 6 μg/ml DAP (Supplementary Table S2) but did not appear in any of the three colonies derived from this population (Supplementary Tables S2, S3). The relatively high number of reads with this SNP, the fact that the same mutation occurred in population A at 10 μg/ml DAP exposure, and the fact that this SNP has been documented in other DAP resistant *S. aureus* strains (Peleg et al., 2012) all suggest that this mutation is real but inhibited growth on plates or was not represented by the small sampling size in the colonies. The SNP at position 1706314 was present in only 8% of population B but occurred in 100% of the reads for two of the three colonies (Supplementary Table S2). Similarly, a mutation present at position 352545 in the ABC transporter permease gene SA0296 occurred in only 5% of the reads for population P (not reaching our cutoff threshold) but was present in 100% of the reads for colony 3 from this population (Supplementary Table S2).

## Discussion

We describe a novel approach to studying the accumulation of genetic mutations that leads to enhanced daptomycin resistance in *S. aureus* N315, a highly DAP-S strain. We employed a continuous culture model using a bioreactor which eliminates the conditions of nutrient limitation and allows bacterial replication to occur unhindered except for antibiotic exposure. Thus, growth of DAP-S strains in a bioreactor continuously exposes the bacteria to a known increasing concentration of daptomycin that constantly challenges the bacterial population and selects for changes related to antibiotic pressure (Toprak et al., 2011, 2013). Additionally, the bioreactor model allows us to continuously monitor bacterial growth in a way that is not possible with hollow fiber or plate dependent methods. The consistently increasing concentration of daptomycin in a bioreactor simulates clinical situations where the bacteria are present in a nutritionally rich environment in pockets where the drug concentration may not reach lethal bactericidal levels. This differential distribution of antibiotic allows some bacteria to persist and selects for DAP-R *S. aureus* strains that are refractory to killing when exposed to higher concentrations of antibiotics.

*S. aureus* populations subjected to increasing concentrations of daptomycin in the bioreactor displayed mutations at loci associated with resistance in clinically and laboratory-derived isolates (Friedman et al., 2006; Howden et al., 2011; Peleg et al., 2012; Bayer et al., 2015; Chen et al., 2015; Kang et al., 2017; Sabat et al., 2018), thereby demonstrating the utility of the bioreactor for studying the *in vivo* and *in vitro* evolution of daptomycin resistance. The three experimental replicates (populations A–C) revealed that the bioreactor yields consistent, reproducible results as evidenced by the repeated evolution of several genetic mutations in more than one population. This supports the hypothesis that daptomycin resistance can occur through multiple routes but evolution converges on and targets a few main pathways.

We also detected SNPs at or near new loci that might contribute to daptomycin resistance, such as a nonsynonymous change in a DNA helicase and a mutation in the noncoding region near DNA-3-methladenine glycosylase (Table 1). These two genes code for proteins that have DNA repair functions and could either assist in mediating mutational load or facilitate mutation depending on gain or loss of function. Mutations in genes that code for proteins involved in oxidative phosphorylation or electron transport might contribute to the altered metabolic activities that accompany the acquisition of antibiotic resistance in *S. aureus* (Somerville and Proctor, 2009; Proctor et al., 2014). In population C, amino acid changes to NADH dehydrogenase (coded for by SA0802), which transfers electrons to menaquinone in the electron transport chain, and MenH (coded for by SA0897), which functions in menaquinone synthesis, could reflect the presence of small-colony variants that we isolated from the bioreactor. These small-colony variants display deficient electron transport chains and are associated with persistent, antibiotic resistant infections (Somerville and Proctor, 2009; Proctor et al., 2014). Metabolic shifts in DAP-R strains were associated with a decrease in the TCA cycle and an increased production of pyrimidines and purines that diverted carbon to the synthesis of cell wall components such as teichoic acid and peptidoglycan (Gaupp et al., 2015). Transcriptomic profiling revealed that the *pur* operon – responsible for purine synthesis – was strongly regulated in DAP-R strains (Müller et al., 2018). Correspondingly, we observed mutations in a transcription factor that controls purine biosynthesis (SA0454) as well as changes in metabolic genes involved in glucose metabolism (SA0219), amino acid synthesis (SA0756), and steroid biosynthesis and degradation (SA0613; Table 1). While serial passaged population P evolved resistance to daptomycin, the SNP in *pgsA* – the only mutation associated with this condition – arose after exposure to the highest concentration of daptomycin. This suggests that transcriptional changes mediated resistance at lower concentrations and illustrates the different results produced by the two culture methods.

Although populations A–C independently acquired many of the same SNPs or mutations at the same loci, the evolutionary landscapes explored were varied and the mutation profiles were distinct (e.g., Figure 2). However, all three populations converged upon a combination of changes – either at *pgsA* and *mprF* or *mprF* and *cls2* – supporting our hypothesis that the presence of only a few key adaptations drives daptomycin resistance in *S. aureus* N315. Both cardiolipin synthase (coded for by *cls2*) and CDP-diacylglycerol-glycerol-3-phosphatidyltransferase (coded for by *pgsA*) are involved in the production of membrane phospholipids (Peleg et al., 2012; Bayer et al., 2013). Thus, modifications to these enzymes could influence daptomycin binding and/or translocation across the membrane via compositional changes or changes to membrane fluidity or charge (Peleg et al., 2012; Bayer et al., 2013). The MprF enzyme lysinylates PG – a major cell membrane component for *S. aureus* – and translocates it to the outer membrane, which influences the charge of the membrane and could ultimately cause repulsion of the DAP-Ca^2+^ complex (Ernst et al., 2009). The presence of mutations in phospholipid biosynthesis genes also represented the unifying characteristic across 21 clinical and 12 laboratory DAP-R isolates, underscoring the importance of a few key genes in providing initial resistance as well as the genomic background capable of surviving increased antibiotic exposure (Peleg et al., 2012). While mutations at *mprF*, *rpoB/C*, and *yycG* (*walK*) loci consistently arose in (varying) sequential order from serial passage experiments (Friedman et al., 2006) and mutations also accumulated sequentially in vancomycin resistant clinical isolates (Mwangi et al., 2007), our results highlight the fact that the evolution of daptomycin resistance does not necessarily rely on a linear addition of genomic mutations. Furthermore, the bioreactor revealed that most mutations arose in the population at an earlier time point than might be recorded from sequencing only the final DAP-R population, a level of detail that our serial passage experiment could not provide. For example, some SNPs present in less than 5% of the population, reached fixation by the end of the experiment. Additionally, the presence of low frequency variants in colonies plated from the DAP-R populations supports the hypothesis that most low-frequency variants were likely real and could be selected for in subsequent generations. The discrepancies between our results and past experiments could reflect strain specific responses or the effects of bioreactor versus plate-dependent growth. Additional bioreactor experiments with different *S. aureus* strains would help to clarify whether the trends we observed are more generalizable. The early appearance of low frequency variants suggests that there could be pre-existing mutations within the population that are selected for at higher concentrations of daptomycin. However, the appearance of additional low frequency variants at later time points, such as in Population C, presents the possibility that mutagenesis induced by drug exposure also plays a role in generating variation, a hypothesis that requires further experimentation as well.

While bioreactor populations consistently evolved pairs of mutations affiliated with daptomycin resistance, not all mutation combinations appear to be created equally as demonstrated by the different fates of mutations at *mprF* and *cls2* loci in populations B and C. At 10 μg/ml, population B contained two SNPs at the *mprF* locus (herein referred to as *mprF* S295L and L341F) represented in approximately equal proportions and two SNPs at the *cls2* locus (herein referred to as *cls2* A23V and L52F) also represented in equal proportions (Figure 2). However, after increasing the antibiotic concentration, *mprF* S295L and *cls2* A23V were represented in almost 100% of the population while *mprF* L341F and *cls2* L52F were essentially extinct (Figure 2). The simultaneous rise of *mprF* S295L and *cls2* A23V with the corresponding extinction of *mprF* L341F and *cls2* L52F suggests that two DAP-R subpopulations existed with the first conferring a selective advantage (Figure 2). Population C displayed the same *cls2* L52F mutation as Population B and an S337L *mprF* mutation (which resides in the same transmembrane domain as position 341). Interestingly, it required an additional 24 h for population C to rebound to the same CFU levels reached by population B at 14 μg/ml. A fourth bioreactor experiment produced a DAP-R population with the same *mprF* and *cls2* mutations as Population C with a nearly identical mutational profile (i.e., SNPs appearing at the same time points with changes in SNP frequencies occurring at the same rates at the same dosages; Supplementary Figure S1) and confirmed the delayed rebound in growth after 14 μg/ml daptomycin exposure. Thus, it is tempting to speculate that the behavior of population C would represent the behavior of the *mprF* L341F/*cls2* L52F subpopulation in population B in the absence of competition. However, validation of fitness differences requires additional experiments that directly test the competition among populations with varying mutation combinations.

## Conclusion

In conclusion, the bioreactor model represents a novel, effective method for recapitulating *in vivo* conditions and inducing DAP-R mutations typically observed in clinical isolates. The bioreactor model reveals that certain key mutations are most likely required to be present to confer daptomycin resistance but the combination of key mutations can vary and mutations do not necessarily arise in a progressive fashion. Furthermore, the competitive advantages of mutation combinations do not appear to be equal even if the mutations arise in the same gene combinations (such as *mprF* and *cls2*). To our knowledge, this study is the first to unveil a possible hierarchical dominance to mutation combinations, although additional experimental confirmation is required. The bioreactor model illustrates that the evolution of DAP-R is a dynamic process, a characteristic often not reflected when examining static cultures or isogenic strains. Tracking the collective evolutionary fates of genomic mutations over the course of antibiotic treatment will assist in identifying early signs of resistance and might provide knowledge of which mutations will be more responsive to treatment.

## Author Contributions

KM, JP, MP, and MM conceived of the study. MM, JL, ZM, VR, and SM maintained the bioreactor and all cultures. ZM performed DNA extractions while RS submitted DNA for sequencing and contributed to data interpretations. MP generated Figure 1. EL-N and MM wrote the manuscript and EL-N performed all whole-genome sequencing analyses. All authors read and approved the final manuscript.

## Conflict of Interest Statement

The authors declare that the research was conducted in the absence of any commercial or financial relationships that could be construed as a potential conflict of interest.

## Footnotes

1 https://www.beiresources.org/

2 https://sourceforge.net/projects/bbmap

## Supplementary Material

FIGURE S1 Mutation profiles of population C and a fourth bioreactor experiment (population D). The frequency of mutations as a function of antibiotic concentration for population C (top) and population D (bottom). Mutations highlighted are SNPs that lead to amino acid changes in MprF and Cls2 and lower frequency variants NADH (NADH dehydrogenase, SA0802), menH (2-succinyl-6-hydroxy-2, 4-cyclohexadiene-1-carboxylate synthase, SA0897), and rpoA (DNA-directed RNA polymerase subunit alpha, SA2023). SNPs are identical between the populations and display the same dynamics (i.e., SNPs appear at the same time and change in frequency at the same rate and at the same dosages).

TABLE S1 SNPs present in the parental DAP-S isolate of N315 in comparison to the NCBI Refseq N315 isolate. These changes were detected in our BEI parental N315 strain and all descendant populations, except for the mutation at position 939485, which is not present in Population C. Annotations associated with noncoding SNPs are for the nearest coding sequence.

TABLE S2 Allele Frequencies of SNPs for populations 6–8, population P and colonies 1–3 derived from population 6 at 6 μg/ml and populations 7 and P at 10 μg/ml daptomycin. Alt, Alternative (SNP) nucleotide frequency; Reads, total number of reads at a position. All concentrations are of daptomycin. No colonies were sequenced from Population C.

TABLE S3 SNPs present in colonies from Population A taken at 6 μg/ml daptomycin and populations B and P at 10 μg/ml daptomycin. Three colonies were sequenced from each population. SNPs present in the ancestral population are not shown.
