# Supplementary figures and images for: Insights Into the Evolution of Staphylococcus aureus Daptomycin Resistance From an in vitro Bioreactor Model
Source: Front Microbiol. 2019 Feb 28;10:345. doi: 10.3389/fmicb.2019.00345 (PMC6413709; doi:10.3389/fmicb.2019.00345)

# Population C

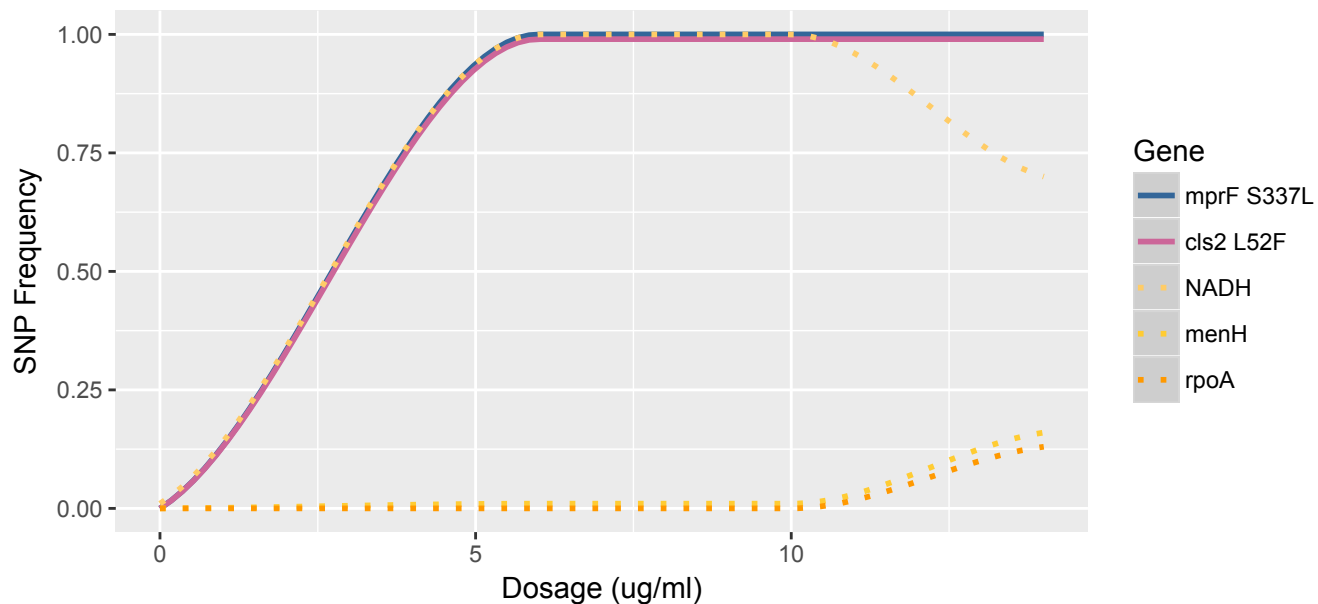

# Population D

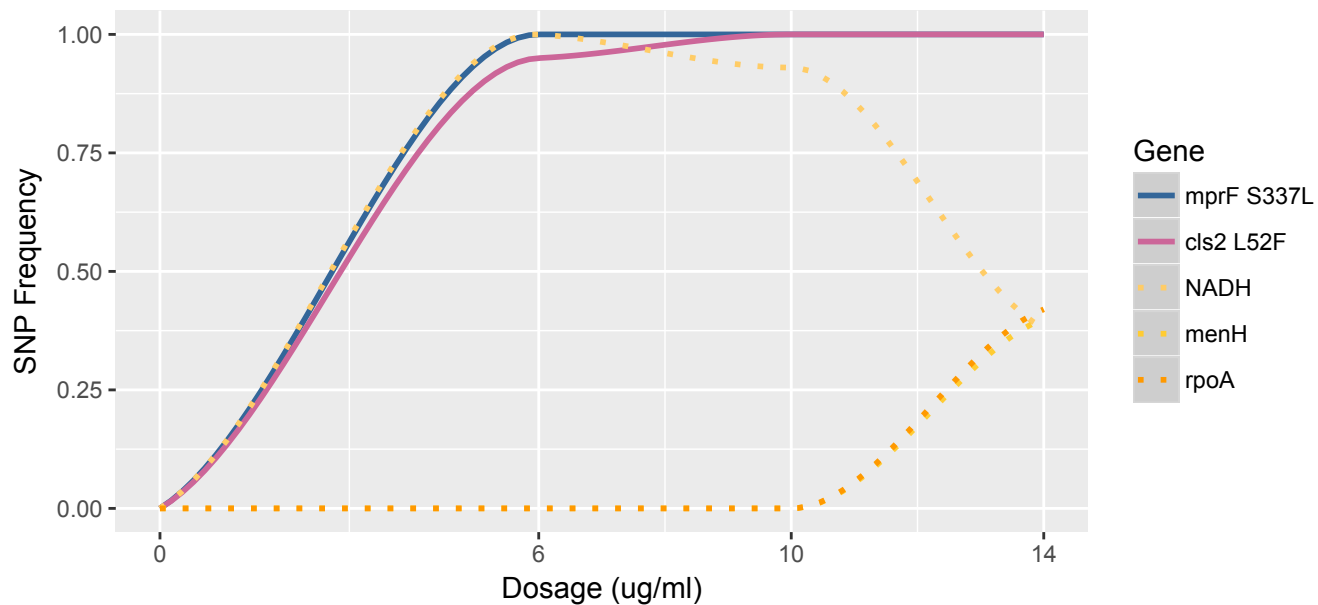

Supplement: FIGURE S1 — Mutation profiles of population C and a fourth bioreactor experiment (population D). The frequency of mutations as a function of antibiotic concentration for population C (top) and population D (bottom). Mutations highlighted are SNPs that lead to amino acid changes in MprF and Cls2 and lower frequency variants NADH (NADH dehydrogenase, SA0802), menH (2-succinyl-6-hydroxy-2, 4-cyclohexadiene-1-carboxylate synthase, SA0897), and rpoA (DNA-directed RNA polymerase subunit alpha, SA2023). SNPs are identical between the populations and display the same dynamics (i.e., SNPs appear at the same time and change in frequency at the same rate and at the same dosages). [file Image_1.pdf]
